# Central Retinal Vein Occlusion Associated With Progestin-Only Contraceptive Implant Use

**DOI:** 10.1155/crop/5558602

**Published:** 2025-07-14

**Authors:** Jan Patrick O. Chu, David I. T. Sia

**Affiliations:** ^1^Department of Ophthalmology, University of Adelaide, Royal Adelaide Hospital, Adelaide, Australia; ^2^Department of Ophthalmology, Flinders University, Flinders Medical Centre, Bedford Park, Australia; ^3^Department of Ophthalmology, Women's and Children's Hospital, North Adelaide, Australia

**Keywords:** central retinal vein occlusion, etonogestrel, progestin-only contraceptive

## Abstract

**Purpose:** The purpose of the study is to present a case of central retinal vein occlusion (CRVO) associated with the use of progestin-only contraceptive implant.

**Methods:** The study is a case report.

**Results:** This is a case of a 34-year-old Caucasian female nonsmoker who presented with sudden-onset painless blurring of vision of the left eye. There were no associated ocular and systemic signs or symptoms. She has no comorbidities. The patient disclosed that she was on the etonogestrel implant, a progestin-only contraceptive, at the time of consult. She was previously diagnosed with a CRVO of the right eye while on the combined hormonal contraceptive in 2017. She had a best corrected visual acuity (BCVA) of 6/6 in both eyes. Anterior segment examinations of both eyes were unremarkable. Fundus examination of the right eye revealed normal findings; however, the fundus exam of the left eye showed a clear media with a healthy looking disc but with sparse dot blot hemorrhages in all retinal quadrants and dilated and tortuous retinal vessels, along with a healthy-looking macula. Macular optical coherence tomography (OCT) did not show any signs of macular edema. Carotid ultrasound, autoimmune, and coagulation panels were all normal. Subsequent visits showed complete resolution of the retinal hemorrhages, and the retinal vasculature returned to normal anatomical configuration. A diagnosis of left CRVO secondary to etonogestrel implant was made.

**Conclusion:** This study recognized that the use of progestin-only contraceptives still has an increased risk of thrombosis. It is essential to consider the mode of administration and duration of use of these contraceptives.

## 1. Introduction

Central retinal vein occlusion (CRVO) is one of the most prevalent retinal vascular diseases with an incidence of 0.8 per 1000 individuals [1]. It is the second most common cause of blindness due to retinal vascular diseases [2].

The pathogenesis of CRVO can be summarized by Virchow's triad: hemodynamic changes, vascular endothelial damage, and hypercoagulable state. It usually manifests with dilated and tortuous retinal veins, diffuse retinal hemorrhages with or without macular edema, and optic disc edema [3, 4]. Risk factors in the elderly are mainly around cardiovascular risks such as advancing age, hypertension, hyperlipidemia, smoking, and diabetes. Other causes include ocular hypertension, inflammatory and infectious diseases, hyperviscosity and hypercoagulable syndromes, mass effect, and drug-related causes [3, 5]. In the young, risk factors are much more diverse compared to the elderly and noncardiovascular-related risks are more common.

Oral contraceptive pills have been associated with the risk of thrombosis and CRVO [5]. The risk of venous thromboembolism is thought to be highest in the combined estrogen/progestin oral contraceptive and lowest in progestin-only parenteral contraceptive options such as intrauterine devices (Mirena) and implants (Implanon, Norplant) [6]. This report describes a rare case of CRVO associated with progestin implant (Implanon) use. As far as we are aware, this has not been described before in the literature.

## 2. Case Report

This is a case of a 34-year-old Caucasian female who presented with sudden-onset painless blurring of vision of the left eye. Past medical history revealed COVID-19 infection in 2022, but otherwise, no other comorbidities. She was a nonsmoker. Past ocular history included laser in situ keratomileusis (LASIK) in both eyes and right CRVO diagnosed in 2017. This was thought to be associated with the use of the combined OCP. A full workup including autoimmune, infective, hyperviscosity, and hypercoagulability screens were negative at that time. The only risk factor was that she was on the combined OCP.

**Figure 1:**
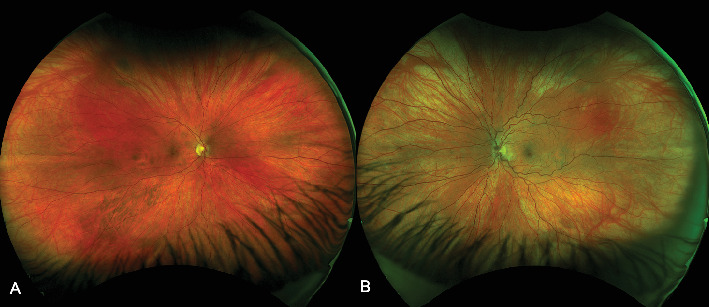
(A) Fundus photo of the right eye showing a normal retina. (B) Fundus photo of the left eye showing dilated and tortuous retinal vessels with sparse dot blot hemorrhages in all retinal quadrants.

**Figure 2:**
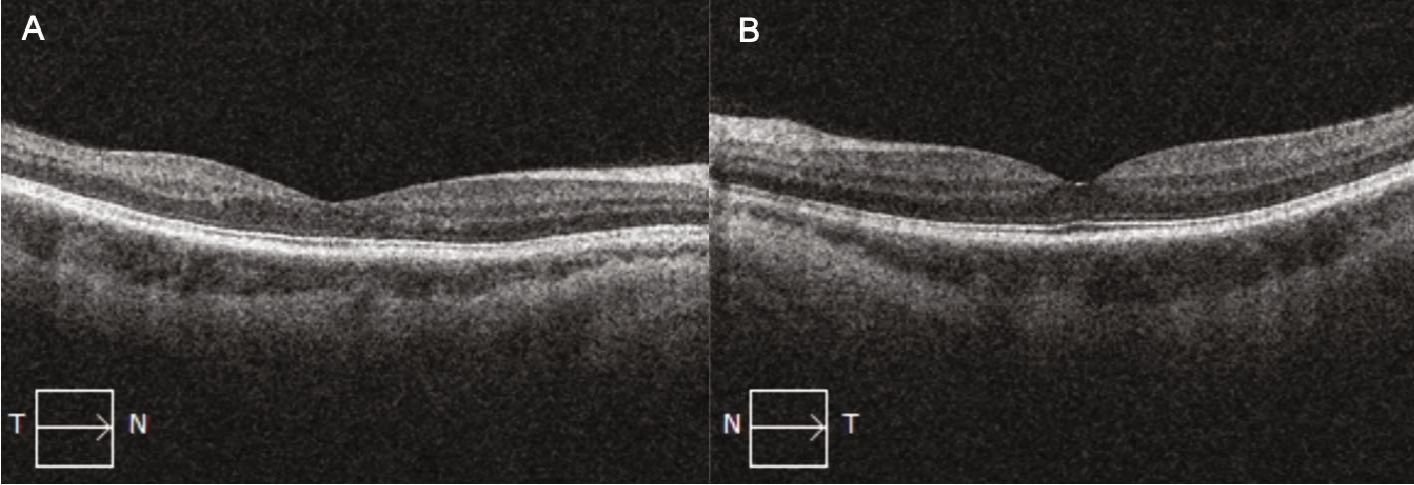
Optical coherence tomographies of the (A) right and (B) left maculas showing a normal foveal contour without intraretinal and subretinal fluid.

On this presentation, she had a best corrected visual acuity (BCVA) of 6/6 in both eyes. Anterior segment examination of both eyes was unremarkable. Fundus exam of the left eye showed sparse dot blot hemorrhages in all retinal quadrants with dilated and tortuous retinal veins (Figure 1). There was no cystoid macular edema (CME) on OCT (Figure 2). Fundus examination of the right eye was normal.

All investigations for autoimmune, infective, hyperviscosity, and hypercoagulable causes were negative. This included antinuclear antibody, double-stranded DNA antibody, rheumatoid factor, anticardiolipin antibody, lupus anticoagulant, antithrombin III activity, protein C and S activities, Factor V Leiden, Factor VIII, prothrombin gene mutation, and serum protein electrophoresis. The patient disclosed that she was on the progestin contraceptive implant, Implanon. Following her diagnosis of right CRVO in 2017, she was switched from the combined OCP to the progestin implant (Implanon), a slow release etonogestrel contraceptive implant, in March 2018. The total duration of implant use was 85 months, and it was removed in May 2024, 2 weeks after her symptoms began in the left eye.

**Figure 3:**
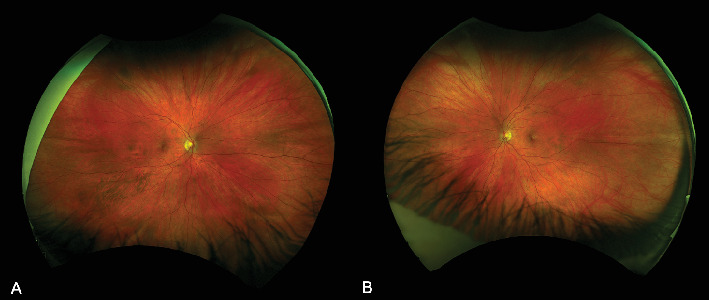
Fundus photos of the (A) right and (B) left eyes 5 months after first presentation showing normal retinas without hemorrhages. Note that the tortuosity and dilatation of the retinal vessels in the left eye have resolved.

**Figure 4:**
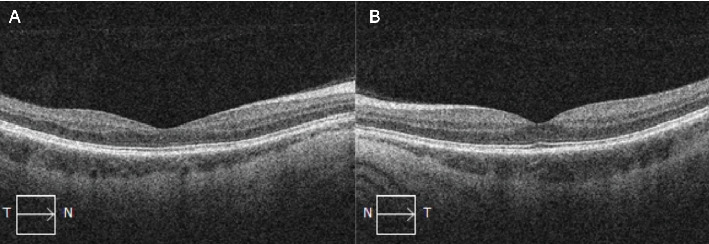
Optical coherence tomographies of the (A) right and (B) left maculas 5 months after first presentation showing no anatomical changes.

**Figure 5:**
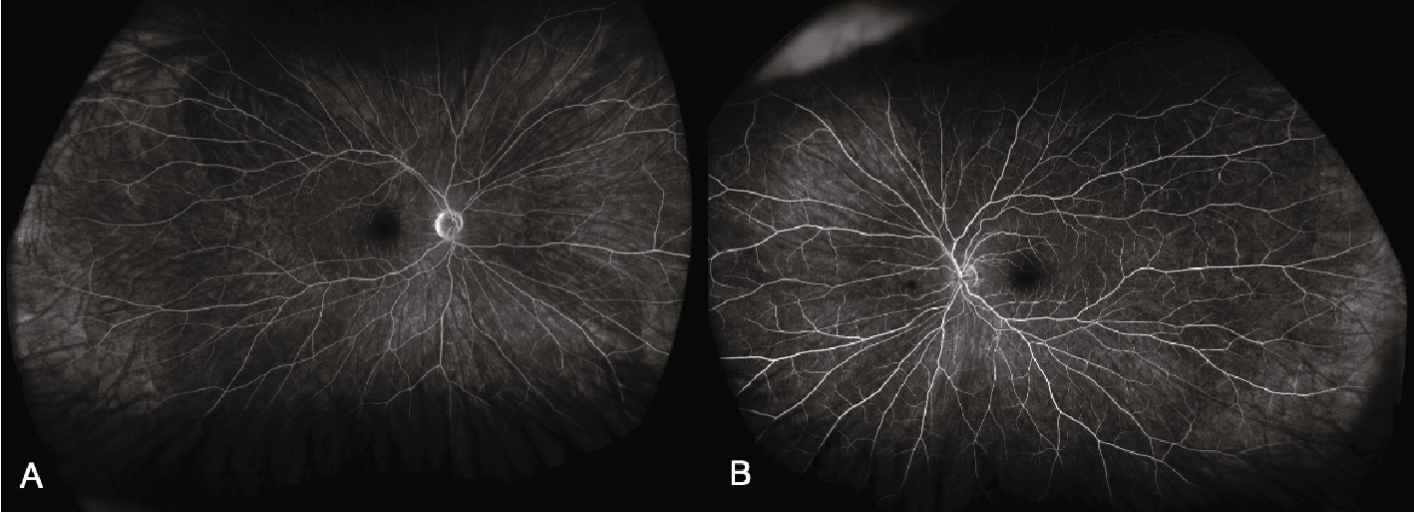
(A, B) Fundus fluorescein angiogram of both eyes showing normal dye transit with no areas of capillary dropout and leakages.

At 1 month, there was complete resolution of retinal hemorrhages, and retinal vasculature returned to normal appearance (Figure 3). Macular OCT of both eyes was normal (Figure 4). Fundus fluorescein angiogram showed normal dye transit in both eyes (Figure 5).

Following complete resolution, the patient was reviewed again in 6 months. No medications were prescribed for her CRVO.

## 3. Discussion

CRVO is typically seen in the aging population [1, 3], with a prevalence rate of 0.1%–0.2% [3]. However, it can also occur in younger individuals. CRVO in the young is defined as CRVO in an individual < 50 years old [4].

Data regarding the pathogenesis and clinical course of CRVO in the young are still limited. However, there are studies that suggest that CRVO patients < 50 years of age have better presenting visual acuities and final visual acuities than patients ≥ 50 years of age [3]. Research also indicates that younger patients typically exhibit less severe clinical manifestations than older patients with CRVO. They are more likely to experience nonischemic CRVO, have a lower occurrence of CME and subretinal fluid (SRF), show thinner central retinal thickness (CRT), and maintain better integrity of the external limiting membrane (ELM), ellipsoid zone (EZ), and retinal pigment epithelium (RPE) [4]. As in this case, our patient presented with only mild retinal changes and had good visual recovery.

The etiological factors are much more diverse in CRVO in the young than in the elderly. Apart from traditional cardiovascular risk factors, other conditions to rule out include hyperviscosity syndromes such as macroglobulinemia, multiple myeloma, leukemia, hereditary spherocytosis, and iron deficiency anemia. Thrombophilic conditions and autoimmune diseases such as hyperhomocysteinemia, elevated antiphospholipid and anticardiolipin antibodies, antithrombin III deficiency, and protein C and S deficiencies should also be excluded in young individuals with CRVO [3].

The use of oral contraceptives is an important risk factor for thrombosis in young female patients [7]. It is known to be a risk factor for cardiovascular and cerebrovascular thrombotic diseases [8]. Our patient was using the combined oral contraceptive at the time of diagnosis of CRVO of the right eye. Although several studies suggest that use of hormonal contraceptives increases the risk of retinal vein occlusion (RVO), the exact mechanism is still unclear [7, 8]. The higher dose of ethinyl estradiol in combined OCPs has been implicated in increasing the risk of venous thromboembolic events, especially when combined with third-generation progestins such as etonogestrel and desogestrel [9].

To decrease the risk of thromboembolism with the combined oral contraceptive pill, progestin-only contraceptives were developed and are thought to pose little risk of thrombosis [10]. Studies have stated that the use of etonogestrel implant is associated with thrombin reduction in the first 6 months. However, it has also been found that progestins may lead to arteriosclerosis due to stimulation of neointimal cellular proliferation [6] and increased distensibility in veins and vasoconstriction in arteries, which could cause a reduction in blood flow [11]. Some studies also report that progestin-only contraceptives, particularly in forms of depots, could elevate the risk of venous thromboembolism due to delivery of a higher dosage and potency of progestin [10]. A meta-analysis of eight observational studies reported that injectable progestins were associated with a twofold increase in venous thromboembolic risk [10].

After conducting a literature review utilizing PubMed and Google Scholar using the keywords progestin, etonogestrel, and central retinal vein occlusion, we did not find any prior reports of CRVO associated with progestin-only contraceptive implant (Implanon).

## 4. Conclusion

Anecdotal reports and observational studies have reported that progestin-only contraceptives, particularly third-generation progestins, still pose a risk for thrombosis. Based on the literature at hand, there is still an increased risk of thrombotic events with the use of progestin-only contraceptive implants. The clinical features in our case were mild, and there was good recovery of vision. Physicians who prescribe this medication and ophthalmologists should be aware of this risk and counsel patients accordingly.

## Data Availability

The data that support the findings of this study are available on request from the corresponding author. The data are not publicly available due to privacy or ethical restrictions.
